# Establishing an itaconic acid production process with *Ustilago* species on the low-cost substrate starch

**DOI:** 10.1093/femsyr/foae023

**Published:** 2024-07-22

**Authors:** Philipp Ernst, Astrid Wirtz, Benedikt Wynands, Nick Wierckx

**Affiliations:** Institute of Bio- and Geosciences IBG-1: Biotechnology, Forschungszentrum Jülich GmbH, Wilhelm-Johnen-Straße, 52428 Jülich, Germany; Institute of Bio- and Geosciences IBG-1: Biotechnology, Forschungszentrum Jülich GmbH, Wilhelm-Johnen-Straße, 52428 Jülich, Germany; Institute of Bio- and Geosciences IBG-1: Biotechnology, Forschungszentrum Jülich GmbH, Wilhelm-Johnen-Straße, 52428 Jülich, Germany; Institute of Bio- and Geosciences IBG-1: Biotechnology, Forschungszentrum Jülich GmbH, Wilhelm-Johnen-Straße, 52428 Jülich, Germany

**Keywords:** *Ustilago cynodontis*, starch, itaconic acid, low-cost substrates, glucoamylase, α-glucosidase

## Abstract

*Ustilago maydis* and *Ustilago cynodontis* are natural producers of a broad range of valuable molecules including itaconate, malate, glycolipids, and triacylglycerols. Both *Ustilago* species are insensitive toward medium impurities, and have previously been engineered for efficient itaconate production and stabilized yeast-like growth. Due to these features, these strains were already successfully used for the production of itaconate from different alternative feedstocks such as molasses, thick juice, and crude glycerol. Here, we analyzed the amylolytic capabilities of *Ustilago* species for metabolization of starch, a highly abundant and low-cost polymeric carbohydrate widely utilized as a substrate in several biotechnological processes. *Ustilago cynodontis* was found to utilize gelatinized potato starch for both growth and itaconate production, confirming the presence of extracellular amylolytic enzymes in *Ustilago* species. Starch was rapidly degraded by *U. cynodontis*, even though no α-amylase was detected. Further experiments indicate that starch hydrolysis is caused by the synergistic action of glucoamylase and α-glucosidase enzymes. The enzymes showed a maximum activity of around 0.5 U ml^−1^ at the fifth day after inoculation, and also released glucose from additional substrates, highlighting potential broader applications. In contrast to *U. cynodontis, U. maydis* showed no growth on starch accompanied with no detectable amylolytic activity.

Abbreviations ITA: Itaconate
DO: Dissolved oxygen
MTM: Modified Tabuchi medium
HPLC: High performance liquid chromatography
OD_600_: Optical density at 600 nm
YEPS: Yeast extract, peptone, sucrose medium
MES: 2-(*N*-morpholino) ethane sulfonic acid
KPI: Key performance indicators
DNS: Dinitrosalicylic acid
PCR: Polymerase chain reaction

## Introduction

In view of the growing world population and the overexploitation of fossil fuels, a transition from the fossil-based to bio-based production of chemicals from renewable resources is indispensable (Stegmann et al. 2020). This has already been successfully done for different carboxylic acids like citric, lactic, succinic, or itaconic acid (Chen and Nielsen 2016, Kuenz and Krull 2018). Itaconic acid belongs to the 12 most promising bio-based platform chemicals defined by the U.S. Department of Energy in 2004 (Werpy and Petersen 2004). It is of particular interest as an alternative for petrochemical-based acrylic and methacrylic acid in the polymer industry (Teleky and Vodnar 2019), but also has a variety of biological activities that makes it relevant in medical and pharmaceutical sectors (Michelucci et al. 2013, Mills et al. 2018, Olagnier et al. 2020).

To date, itaconic acid is commercially produced using *Aspergillus terreus* achieving titers up to 160 g l^−1^ and yields of up to 0.58 g_ITA_ g_GLC_^−1^ in pulsed batch fermentations on glucose (Krull et al. 2017). However, to be competitive with the petrochemical sector, production costs need to be further reduced, which are significantly influenced by the feedstock used (Saur et al. 2023). One alternative feedstock offering potential cost reductions is the polysaccharide starch. Starch is highly abundant in nature and the most widely utilized substrate for biofuel production. It can be obtained from a variety of agricultural raw materials such as potatoes, wheat, and corn for industrial production in relatively high purity, simplifying downstream processing (Celińska et al. 2021, Singh et al. 2022). Its metabolization entails the liquefaction by an α-amylase and subsequent saccharification into glucose by a glucoamylase (Ebrahimian et al. 2022). Since the increased cultivation of starch-containing plants for industrial purposes can be negatively perceived in public opinion, there is a growing interest in utilizing starchy side streams from food processing industry (Jagadeesan et al. 2020, Kumar et al. 2023, Rodriguez-Martinez et al. 2023). The usage of such industrial side- and waste streams not only reduces production costs, it also makes it possible to achieve the circular bioeconomy concept without compromising food security (Leong et al. 2021). Since *A. terreus* is highly sensitive to medium impurities, the use of such more complex substrates requires pretreatment in order to remove trace elements, which is in addition to the laborious handling and difficult oxygenation due to its filamentous growth one major drawback of this production organism (Klement and Büchs 2013). Therefore, we focus on the basidiomycetes *Ustilago maydis* and *Ustilago cynodontis* as alternative natural itaconate producing strains. Both strains have already been metabolically and morphologically engineered to maintain yeast-like growth and to enable high-level itaconate production at the maximum theoretical yield of 0.72 ± 0.02 g_ITA_ g_GLC_^−1^ in the production phase with a constant glucose feed (Hosseinpour Tehrani et al. 2019a,b, Becker et al. 2021). The robustness of *Ustilago* species to medium impurities and its repertoire of hydrolytic, secretory enzymes makes it a promising candidate for itaconate production based on more complex substrate in a consolidated process (Mueller et al. 2008, Becker et al. 2023). This has already been demonstrated by the use of the untreated, sucrose-containing side streams molasses and thick juice from sugar industry as well as crude glycerol from biodiesel production as feedstock for *Ustilago*-based itaconate production (Helm et al. 2023, Niehoff et al. 2023, Saur et al. 2023). Furthermore, activation of intrinsic xylanases, cellulases, and pectinases enabled degradation of the plant cell wall components hemicellulose, cellulose, and pectin (Geiser et al. 2016, Müller et al. 2018, Stoffels et al. 2020), even though direct usage of lignocellulosic biomass usually requires costly pretreatment to destroy its recalcitrant structure (Regestein et al. 2018).

Here, we performed a proof-of-concept study on the amylolytic potential of *U. maydis* and *U. cynodontis* for the direct utilization of potato starch as a feedstock for itaconate production in a consolidated bioprocess.

## Material and methods

### Chemical and strains

The chemicals used in this study were obtained from Sigma-Aldrich (St. Louis, USA), Thermo Fisher Scientific (Waltham, USA), or VWR (Radnor, USA) and were of analytical grade.

All strains used in this work are listed in Table 1.

**Table 1.** *Ustilago* strains used in this study.

Strain designation	Resistance	Reference
*U. maydis* MB215 ∆*cyp3* ∆MEL ∆UA ∆*dgat* ∆*P_ria1_::P_etef_* ∆*fuz7 P_etef_mttA*_K14 (198)	Hyg^R^	Becker et al. (2021)
*U. cynodontis* NBRC9727 ∆*fuz7* ∆*cyp3 P_etef_mttA P_ria1_ria1* (223)	Hyg^R^, Cbx^R^	Hosseinpour Tehrani et al. (2019b)
*U. cynodontis* NBRC9727 ∆*fuz7* ∆*cyp3 P_etef_mttA P_ria1_ria1 P_etef_α-amylase* (2699)	Hyg^R^, Cbx^R^, G418^R^	This study
*U. cynodontis* NBRC9727 ∆*fuz7* ∆*cyp3 P_etef_mttA P_ria1_ria1* ∆*UMAG_04 064* (2700)	Hyg^R^, Cbx^R^, G418^R^	This study
*U. cynodontis* NBRC9727 ∆*fuz7* ∆*cyp3 P_etef_mttA P_ria1_ria1 P_etef_cyp3* ∆*UMAG_02 740* (2701)	Hyg^R^, Cbx^R^, G418^R^	This study

### Media and culture conditions

*Ustilago* strains were grown in YEPS medium containing 10 g l^−1^ yeast extract, 10 g l^−1^ peptone, and 10 g l^−1^ sucrose. For growth and production experiments, *Ustilago* strains were cultured in 100 mM MES pH 6.5 buffered screening medium (MTM) according to Geiser et al. (2014) with either glucose (10 g l^−1^) or gelatinized potato starch (10 or 50 g l^−1^). The medium also contained 15 mM NH_4_Cl, 0.2 g l^−1^ MgSO_4_·7H_2_O, 0.01 g l^−1^ FeSO_4_·7H_2_O, 0.5 g l^−1^ KH_2_PO_4_, 1 ml l^−1^ vitamin solution, and 1 ml l^−1^ trace element solution. The vitamin solution contained (per liter) 0.05 g d-biotin, 1 g d-calcium pantothenate, 1 g nicotinic acid, 25 g myo-inositol, 1 g thiamine hydrochloride, 1 g pyridoxol hydrochloride, and 0.2 g para-aminobenzoic acid. The trace element solution contained (per liter) 1.5 g EDTA, 0.45 g of ZnSO_4_·7H_2_O, 0.10 g of MnCl_2_·4H_2_O, 0.03 g of CoCl_2_·6H_2_O, 0.03 g of CuSO_4_·5H_2_O, 0.04 g of Na_2_MoO_4_·2H_2_O, 0.45 g of CaCl_2_·2H_2_O, 0.3 g of FeSO_4_·7H_2_O, 0.10 g of H_3_BO_3_, and 0.01 g of KI. Cultivations were performed in 500 ml shaking flasks with a filling volume of 50 ml (d = 25 mm, *n* = 200 rpm, T = 30°C, and Φ = 80%). For growth and production experiments, main cultures were inoculated to an OD_600_ of 0.5 with precultures grown in the same media.

The DASGIP Bioblock system (Eppendorf, Germany) was used to conduct the batch fermentations, which were controlled using the Eppendorf DASware® control software (Eppendorf, Germany). Vessels with a total volume of 2.3 l and a working volume of 1.0 l were used. The cultivations were performed in batch medium according to Geiser et al. (2014) as described above. The medium also contained 1 g l^−1^ yeast extract (Merck Millipore, Germany) and either 100 g l^−1^ gelatinized potato starch or 200 g l^−1^ α-amylase pretreated potato starch. To avoid clumping of starch during autoclaving, a slurry was prepared beforehand by mixing starch in hot water. For the α-amylase pretreatment, a 1% (v/v) solution of heat-stable *Bacillus licheniformis* α-amylase (Sigma-Aldrich) was added through the septum and incubated for ~2 h. The slurry was maintained at 80°C during the pretreatment. Afterwards, the temperature was adjusted to 30°C, and the remaining medium compounds were added through the septum. Finally, the bioreactor was inoculated to a OD_600_ of 0.75 from an preculture grown in screening medium according to Geiser et al. (2014) containing 15 mM NH_4_Cl, 100 mM MES pH 6.5, and 50 g l^−1^ gelatinized potato starch. The pH was automatically controlled by adding 5 M NaOH or 1 M HCl, while the DO was maintained at 30% using a cascade that involved agitation at 800–1200 rpm (0%–40% DOT controller output), air flow at 1–2 vvm (40%–80% DOT controller output), and oxygen at 21%–100% oxygen (80%–100% DOT controller output). Additionally, 0.5 ml of Antifoam 204 (Sigma-Aldrich) was added at the beginning of the cultivation and every 24 h thereafter.

### Analytical methods

Identification and quantification of products and substrates present in the supernatants were conducted using a high performance liquid chromatography (HPLC) 1260 Infinity system (Agilent, Waldbronn, Germany) equipped with an ISERA Metab AAC column 300 mm × 7.8 mm column (ISERA, Germany). Separation was achieved through isocratic elution at a flow rate of 0.6 ml min^−1^ and a temperature of 40°C, employing 5 mM sulfuric acid as a solvent. Detection involved a diode array detector at 210 nm and a refraction index detector. Analytes were identified based on their retention time compared to corresponding authentic standards, and data analysis was performed using the Agilent OpenLAB Data Analysis—Build 2.200.0.528 software (Agilent). Ammonium concentrations in culture samples were determined using the colorimetric method outlined by Willis et al. (1996). In this method, 50 µl culture supernatant was mixed with 1 ml reagent (8 g sodium salicylate, 10 g trisodiumphosphate, and 0.125 g sodium nitroprusside), followed by rapid addition of 250 µl hypochlorite solution. After color development, the absorbance was measured at 685 nm using cuvettes and a spectrophotometer. Ammonium concentrations were calculated using an ammonium calibration. Cell densities were quantified by measuring the optical density at a wavelength of 600 nm (OD_600_) using cuvettes and a spectrophotometer. Samples were diluted appropriately with the respective medium to ensure measurement within the linear range of the photometer, falling between absolute values of 0.2 and 0.4. SDS-PAGE was performed according to the manufacturer’s instructions using NuPAGE 12% Bis-Tris precast gels and MOPS as the running buffer. A volume of 10 µl of each sample and 5 µl of the protein ladder were loaded onto the gel. After electrophoresis, the gels were stained with Coomassie (Gel Code Blue). For the detection of residual starch in culture samples, 100 µl of clarified culture broth was combined with 100 µl of Lugol’s iodine solution. A volume of 150 µl of the iodine-treated sample was transferred to a transparent flat-bottomed 96-well microplate and the absorbance at 580 nm was measured using microplate reader. The amount of starch was calculated using standard curves of starch.

### Determination of amylolytic enzyme activity

#### Commercial α-amylase assays—blue and red starch polymers

The assays were performed according to the manufacturer’s instructions. Briefly, covalently attached dyes were liberated from starch polymers at a speed proportional to the α-amylase activity. The concentration of the free dyes were detected spectrophotometrically at 580 nm and converted into α-amylase activities using manufactures calibration. Both substrates are exclusively designed for measuring α-amylase activity, as no other enzyme can act upon the substrates due to the cross-linkages and the large dye molecules.

#### Decrease of iodine-binding starch material according to Xiao et al. (2006)

A volume of 40 µl culture supernatant was combined with 40 µl 0.1 M phosphate buffer pH 7.0 containing 0.2% gelatinized potato starch and incubated at 30°C for 30 min. Reactions were terminated by adding 20 µl 1 M HCl. Following termination, 100 µl of Lugol’s iodine solution was added. A volume of 150 µl of the iodine-treated sample was transferred to a transparent flat-bottomed 96-well microplate and the absorbance at 580 nm was measured using a microplate reader. The amount of disappeared starch was calculated using standard curves of starch. The activity was defined as the amount of culture supernatant required for the disappearance of an average of 1 µg of iodine-binding starch material per ml and minute in the assay reaction.


\begin{eqnarray*}
\mathrm{\mathrm{ \mu}} \mathrm{ g}\,\,{\mathrm{ min}^{ - 1}}\mathrm{ m{L}^{ - 1}} = \frac{{{\mathrm{A}}580{\mathrm{\,\,nm\,\,\mathrm{ control}\,\,}} - {\mathrm{\,\,A}}580{\mathrm{\,\,nm\,\,\mathrm{ sample}}}}}{{{\mathrm{A}}580{\mathrm{\,\,nm\,\,}}\left( {{\mathrm{\mu g\,\,\mathrm{ starch}}}} \right){\mathrm{\,\, * \,\,\mathrm{ t}\,\, * \,\,\mathrm{ v}}}}}{\mathrm{ * \ \mathrm{ D}}}.
\end{eqnarray*}

A580 nm control: absorbance of starch without the addition of culture supernatant (-).
A580 nm sample: absorbance of starch digested with culture supernatant (-).
A580 nm (µg starch): absorbance per 1 µg of starch as derived from the standard curve (µg^−1^).
t: incubation time (min).
v: volume of culture supernatant used (ml).
D: dilution factor (-).

#### Increase of reducing sugar concentration according to Miller (1959)

A volume of 40 µl culture supernatant was combined with 40 µl 0.1 M phosphate buffer pH 7.0 containing 0.2% gelatinized potato starch and incubated at 30°C for 30 min. Reactions were terminated by adding 120 µl of dinitrosalicylic acid (DNS) reagent and boiling reaction mixtures for 15 min at 95°C. A volume of 150 µl of the DNS-treated sample was transferred to a transparent flat-bottomed 96-well microplate and the absorbance at 540 nm (*A*540) was measured using a microplate reader. The activity was defined as the amount of culture supernatant required for the release of 1 µg or 1 µmol glucose per ml and minute in the assay reaction.


\begin{eqnarray*}
\mu \mathrm{ g}\,\,{\mathrm{ min}^{ - 1}}\mathrm{ m}{\mathrm{ L}^{ - 1}} &=& \frac{{{\mathrm{A}}580{\mathrm{\,\,nm\,\,\mathrm{ control}\,\,}} - {\mathrm{\,\,A}}580{\mathrm{\,\,nm\,\,\mathrm{ sample}}}}}{{{\mathrm{A}}580{\mathrm{\,\,nm\,\,}}\left( {{\mathrm{\mu \mathrm{ g}\,\,\mathrm{ glucose}}}} \right){\mathrm{\,\, * \,\,t\,\, * \,\,v}}}}{\mathrm{* \ D}}.\\
\mathrm{ U}\,\,{\mathrm{ mL}^{ - 1}} &=& \mu \mathrm{ mol}\,\,{\mathrm{ min}^{ - 1}}{\mathrm{ mL}^{ - 1}}\\
&=& \frac{{{\mathrm{A}}580{\mathrm{\,\,nm\,\,\mathrm{ control}\,\,}} - {\mathrm{\,\,A}}580{\mathrm{\,\,nm\,\,\mathrm{ sample}}}}}{{{\mathrm{A}}580{\mathrm{\,\,nm\,\,}}\left( {{\mathrm{\mu mol\,\,\mathrm{ glucose}}}} \right){\mathrm{\,\, * \,\,t\,\, * \,\,v}}}}{\mathrm{ * \ D}}.
\end{eqnarray*}

A580 nm control: absorbance of sample without the addition of culture supernatant (-).
A580 nm sample: absorbance of sample digested with culture supernatant (-).
A580 nm (µg glucose): absorbance per 1 µg of glucose as derived from the standard curve (µg^−1^).
A580 nm (µmol glucose): absorbance per 1 µmol of glucose as derived from the standard curve (µmol^−1^).
t: incubation time (min).
v: volume of culture supernatant used (ml).
D: dilution factor (-).

### LC-MS/MS

For in-gel digestion, samples were prepared according to Lavigne et al. (2009). Briefly, the decolorization of the gel pieces was carried out in 3 × 350 µl in NH_4_HCO_3_ in 50% acetonitrile in 1.5 ml Eppendorf LoBind tubes, and were incubated 30 min, gently shaken by 300 rpm at room temperature. For tryptic digestion the vacuum dried slices were treated with the Trypsin Singles Proteomics Grade Kit (Sigma-Aldrich) according to the manufacturer’s instructions for in-gel digestion preparation without the reduction and alkylation step. After the incubation at 37°C overnight, each of the samples were submerged in a new LoBind tube with 100 µl 20 mM NH_4_HCO_3_ and were sonicated for 20 min. This step was carried out twice with 50 µl of 5% formic acid in 50% acetonitrile. The samples concentrated in the SpeedVac were taken up in 50 µl 0.1% formic acid and were stored at −20°C before the next sample preparation step. The StageTipping desalting step was carried out as described by Rappsilber et al. (2007). The tryptic peptide samples were stored at −20°C until use for MS measurements.

For analysis of entire supernatants, protein solutions of 150 µl were mixed with 0.25 volume of 100% (w/v) TCA, incubated for 30 min on ice and the precipitated proteins were sedimented for 15 min at 16 100 *g* and 4°C. The supernatants were removed and the precipitated proteins washed twice in 0.5 ml of ice-cold acetone. After centrifugation again (15 min, 16 100 *g*, 4°C), the supernatants were discarded, the pellets were air-dried, and the proteins dissolved in 100 µl Trypsin Reaction Buffer buffer (40 mM NH_4_HCO_3_ pH 8.2, 9% acetonitrile). Before analyzing the protein samples with LC-MS/MS, they were digested using trypsin to cleave the proteins into peptides using the Trypsin Singles, Proteomics Grade Kit (Sigma-Aldrich) according to the manufacturer’s instructions. For this, 1 µg trypsin and 1 µl of Trypsin Solubilization Reagent (contain 1 mM HCl) was added up to 100 µg of protein in a total volume of maximum 100 µl. The tryptic digest was performed at 37°C in an overnight reaction (18 h). Then, 10 µl of 10% formic acid in HPLC-grade water was added to inactivate the protease. The StageTipping desalting step was carried out as described by Rappsilber et al. (2007). The sample was dried and resuspended in 30 µl solvent A (0.1% formic acid in H_2_O). Subsequent LC-MS/MS analysis of trypsin-digested samples was done as already described previously by Hünnefeld et al. (2021). The IDA data were processed with ProteinPilot (5.02, Sciex) using the Paragon algorithm for protein identification and for building an ion library. This data was then compared with a database consisting of proteins from *U. maydis* 521 and *U. cynodontis* NBRC9727.

### Plasmid cloning and strain engineering

Plasmids were constructed via Gibson assembly (Gibson et al. 2009) using the NEBuilder HiFi DNA Assembly Cloning Kit [New England Biolabs (NEB), Ipswich, MA, USA]. The DNA oligonucleotides were purchased from Eurofins Genomics (Ebersberg, Germany), and Q5 High-Fidelity DNA Polymerase (NEB) was used as the polymerase. Table 2 and Table S1 provide details on the plasmids and primers used. Competent *Escherichia coli* DH5α or PIR2 cells were used for standard cloning and plasmid maintenance, following the protocols described in Sambrook and Russell (2006). Plasmids were confirmed through polymerase chain reaction (PCR), restriction analysis, or sequencing. The protocols described in Brachmann et al. (2004) were used for the generation of protoplasts, transformation, and isolation of genomic DNA of *Ustilago* strains. To integrate *P_etef_amyA* randomly into the genome, the plasmid was linearized with Bgll. For the deletion of UMAG_04064 and UMAG_02740, homologous recombination with 1000 bp flanking regions including a geneticin G418 resistance cassette were used. Successful integration and deletion were confirmed by PCR.

**Table 2.** Plasmids used in this study.

Plasmid	Description	Reference
pJET1.2/blunt	Ori ColE1, Amp^R^	Thermo Scientific, Germany
*P_etef_gfp*_G418	Constitutive *P_etef_* promotor, *gfp* gene, G418^R^	Przybilla, Roxense BioSC
*P_etef_amyA*_G418	Constitutive *P_etef_* promotor, *α-amylase* gene from *U. cynodontis* NBRC9727, G418^R^	This study
∆*UMAG_04 064*_G418	Deletion of UMAG_04 064 in *U. cynodontis* ITA MAX pH, G418^R^	This study
∆*UMAG_02 740*_G418	Deletion of UMAG_02 740 in *U. cynodontis* ITA MAX pH, G418^R^	This study

## Results and discussion

### Genetic inventory of amylolytic enzymes encoded in *Ustilago* species

The genome of *U. maydis* 521 encodes four putative amylolytic enzymes which cleave α-1,4 and α-1,6 bonds between glucose molecules (Table 3). *Ustilago maydis* 521 was taken as a reference because this strain is fully sequenced and annotated (Kämper et al. 2006) (Refseq assembly GCF_000328475.2). A tblastn analysis (Altschul et al. 1997, Gertz et al. 2006) of these protein sequences against the whole-genome shotgun contigs database identified putative orthologues of all enzymes in *U. cynodontis* NBRC9727 with protein sequence similarities ranging between 66.2% and 87.3% (Table 3). Putative orthologues of all enzymes were also identified in *U. maydis* MB215. Interestingly, while high protein similarities were found for the α-amylase and α-glucosidase, lower similarities were detected for the maltase and glucoamylase.

**Table 3.** Putative enzymes present in *U. maydis* 521 cleaving α-1,4 and α-1,6-bonds between glucose molecules.

						Protein similarity (%)		GenBank number
Enzyme	EC number	Action	*U. maydis* 521 gene	CAZy family	Reference	*U. maydis* MB215	*U. cynodontis* NBRC9727	*U. cynodontis* NBRC9727
α-amylase	3.2.1.1	α-1,4 bonds are cleaved within starch to release α-dextrins such as G2, G3, G6, and G7 maltooligomers	UMAG_02300 (putative)	GH13	Couturier et al. (2012, Kretschmer et al. (2017)	99.8	87.3	CAKMXY010000005, region 1 296 343 to 1 297 953
Glucoamylase	3.2.1.3	α-1,4 and α-1,6 bonds are cleaved from the nonreducing ends of starch to release glucose	UMAG_04064 (putative)	GH15	Couturier et al. (2012, Kretschmer et al. (2017	26.5	66.2	CAKMXY010000014, region 83 686 to 85 185
Maltase	3.2.1.20	α-1,4 bonds are cleaved in maltose to release glucose	UMAG_15026 (putative)	GH13	Couturier et al. (2012)	63.1	79.9	CAKMXY010000012, region: 816571.818319
α-glucosidase	3.2.1.20	α-1,4 bonds are cleaved in maltose to release glucose; preferentially α-1,4 bonds are cleaved from the nonreducing ends of starch to release glucose	UMAG_02740 (putative)	GH13 & GH31	Mueller et al. (2008), Couturier et al. (2012)	98.7	73.3	CAKMXY010000008, region 881 932 to 885 099

### Shake flask cultivations on potato starch as a sole carbon source

**Figure 1.**
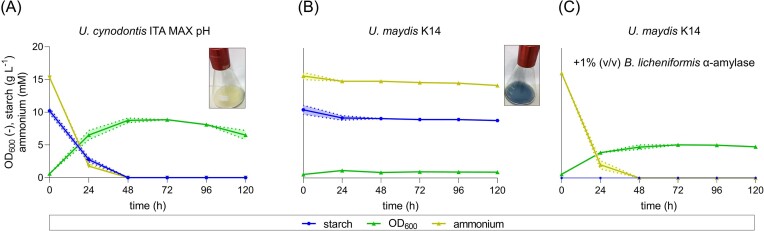
Shake flask cultivations of *U. cynodontis* ITA MAX pH and *U. maydis* K14 on gelatinized potato starch (A and B) and α-amylase pretreated potato starch (C) as a sole carbon source. Shake flask cultivations were performed in MTM medium containing 15 mM NH_4_Cl, 100 mM MES pH 6.5, and 10 g l^−1^ gelatinized potato starch. The mean values with standard deviation of two independent biological replicates are shown.

In order to test the expression of these genes *in vivo* in axenic cultures, cultivations of two previously engineered itaconate-overproducing *Ustilago* strains, *U. cynodontis* ITA MAX pH (Hosseinpour Tehrani et al. 2019b) and *U. maydis* K14 (Becker et al. 2020) were performed with potato starch as a sole carbon source. To avoid the energy-intensive initial gelatinization step, the first cultivations were performed with raw starch powder. However, no growth was detected for either strain (data not shown). Consequently, gelatinized starch was applied in the subsequent cultivations (Fig. 1).

Significant differences between *U. cynodontis* ITA MAX pH and *U. maydis* K14 were observed in terms of growth and starch degradation. Whereas *U. cynodontis* ITA MAX pH reached an optical density of 8.9 ± 0.2 and utilized all starch and ammonium within the first 48 h (Fig. 1A), *U. maydis* K14 showed almost no growth and no decrease of starch or ammonium (Fig. 1B), despite having the same genes present in the genome. Apparently, these genes are not expressed in *U. maydis* K14 under the applied conditions. The basal expression of amylolytic enzymes may not be sufficient to sense the presence of starch. It may also be possible that there are different triggers or even different mechanisms regulating the expression of the amylolytic enzymes between *U. maydis* and *U. cynodontis*, maybe also related to pH. However, *U. maydis* K14 was able to grow on α-amylase pretreated starch up to OD_600_ values of 5.1 ± 0.1 (Fig. 1C), which is consistent with previous literature (Kretschmer et al. 2017).

### Lab-scale fermentations on gelatinized and pretreated potato starch as a sole carbon source

Based on the obtained results, the cultivations were scaled up from shake flasks to bioreactors to analyze itaconate production on starch under more industrially relevant conditions.

**Figure 2.**
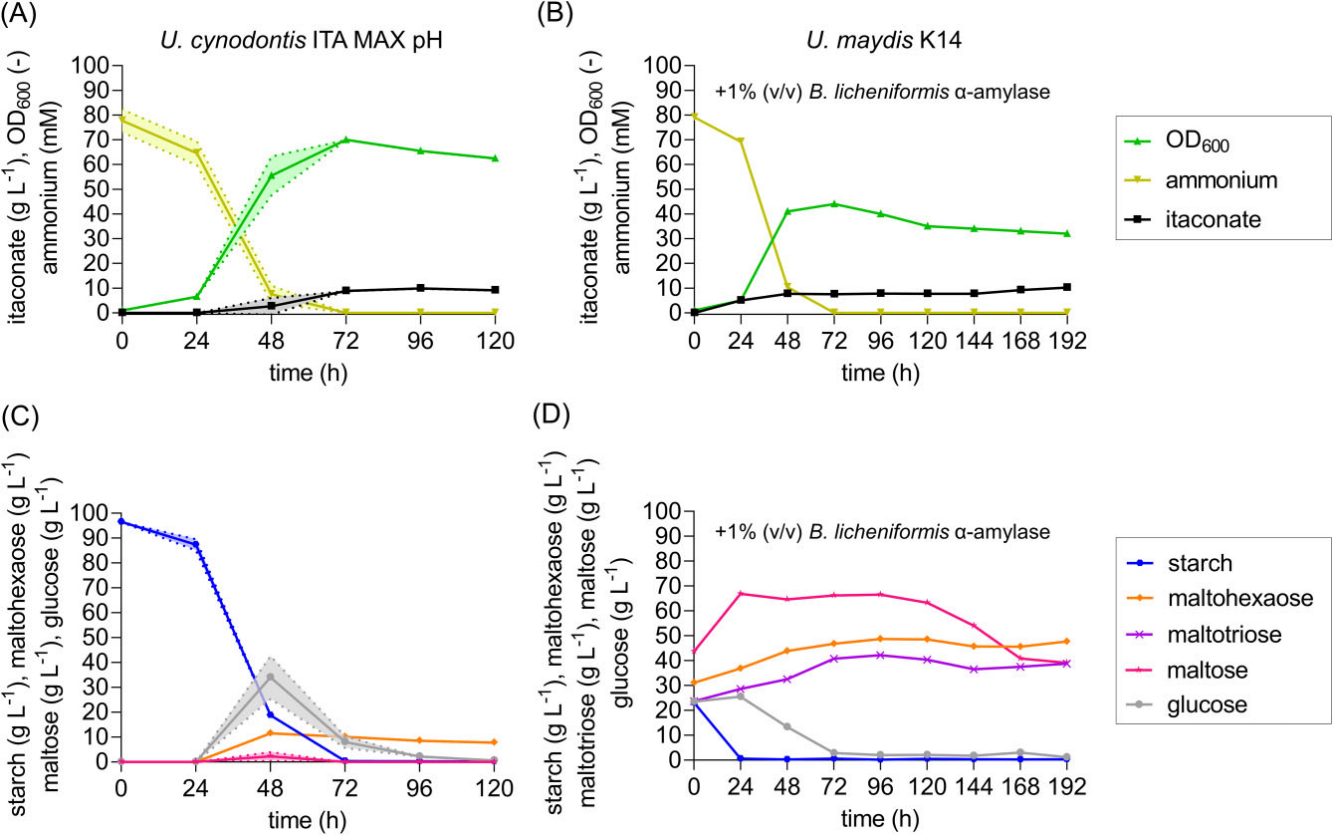
High-density batch fermentations on gelatinized potato starch (A and C) and α-amylase pretreated potato starch (B and D) as a sole carbon source. Concentration of potato starch (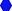), maltohexaose (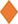), maltotriose (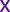), maltose (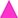), glucose (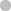), OD_600_ (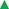), ammonium (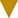), and itaconate (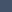) during fermentation in a bioreactor containing batch medium with 75 mM NH_4_Cl and either 100 g l^−1^ gelatinized potato starch (A and C) or 200 g l^−1^ α-amylase pretreated potato starch (B and D). The pH was controlled at pH 3.6 by automatic titration with 5 M NaOH. The mean values with standard deviation of two independent biological replicates are shown for (A) and (C), while (B) and (D) show the values of a single representative culture.

In the first 72 h of the *U. cynodontis* ITA MAX pH fermentations on starch, the cell densities increased up to OD_600_ values of ~70 ± 1 (Fig. 2A), and starch was converted to sugar mono- and maltooligomers (Fig. 2C), which was in line with the shake flask experiments. Starch was mainly hydrolyzed to glucose with a high transient accumulation of ~34 ± 8.6 g l^−1^ reached after 48 h. In addition, 11.6 ± 0.6 g l^−1^ of maltohexaose as well as small amounts of maltose (2.3 ± 1.8 g l^−1^) were detected. Glucose and maltose were constantly metabolized until their complete depletion, whereas the concentration of maltohexaose remained almost constant until the end of the cultivation (7.8 ± 0.9 g l^−1^). This clearly indicates the inability of *U. cynodontis* ITA MAX pH to utilize maltohexaose. In total, this fermentation resulted in the production of 9.1 ± 1.3 g l^−1^ itaconate at a rate of 0.08 ± 0.01 g l^−1^ h^−1^ and a yield of 0.1 ± 0.01 g_ITA_ g_starch_ (unmetabolized sugars not accounted in the yield calculation). These key performance indicators (KPIs) are significantly lower than the ones achieved on pure glucose as a substrate (Hosseinpour Tehrani et al. 2019a). Together with the carbon loss due to unmetabolized maltohexaose, this clearly emphasizes the need of optimization, for instance by overproduction of additional amylolytic enzymes. Such an approach was already successfully applied for production of itaconate from liquefied corn starch with *A. terreus* CICC 40205. While the wildtype strain produced ~14 g l^−1^ itaconate, heterologous overexpression of a glucoamylase gene from *Aspergillus niger* increased production to ~60 g l^−1^ (Huang et al. 2014). Other native itaconate producers such as the thermotolerant *A. terreus* BD strain and *A. niveus* MG183809 also achieve higher KPIs on starch compared to *U. cynodontis* ITA MAX pH. For example, the *A. terreus* BD strain produced 41.1 g l^−1^ itaconate at a rate of 0.19 g l^−1^ h^−1^ and a yield of 0.27 g_ITA_ g_food waste_ during batch fermentation on enzymatic hydrolysates of food waste (potato, rice, and noodles) (Narisetty et al. 2021). *Aspergillus niveus* MG183809 was found to produce itaconate from several starch-containing substrates including wheat flour, corn starch, and sweet potato waste, with the highest itaconate titer of 15.7 g l^−1^ obtained on corn starch (Gnanasekaran et al. 2018). According to a study from Bafana et al. (2019), the recently isolated *A. terreus* C1 strain resulted in the production of 29.7 g l^−1^ itaconate at a rate of 0.21 g l^−1^ h^−1^ and a yield of 0.18 g_ITA_ g_potato starch_ during 3 l batch fermentation on potato starch.

In comparison to *U. cynodontis* ITA MAX pH, the *U. maydis* K14 fermentation on pretreated starch accumulated less biomass with an OD_600_ of 44 after 72 h. Due to the α-amylase pretreatment, most of the starch was already converted to sugar mono- and maltooligomers in the beginning of the fermentation, with a complete hydrolysis obtained after 24 h. The detected glucose concentration was lower than that during the *U. cynodontis* ITA MAX pH fermentation reaching a maximum of 25.5 g l^−1^ after 24 h, which was fully consumed after 72 h. In contrast, higher concentrations of maltose (66.9 g l^−1^), maltotriose (42.2 g l^−1^), and maltohexaose (48.7 g l^−1^) were detected. The latter two remained almost constant until the end of the cultivation. The maltose concentration started to decline after ~120 h, however, a large amount of this disaccharide remained in the final culture supernatant as well. In total, this fermentation resulted in the production of 10.2 g l^−1^ itaconate, while the productivity was reduced to 0.05 g l^−1^ h^−1^ compared to previous fermentation of *U. cynodontis* ITA MAX pH due to the longer fermentation time of 192 h. The yield achieved with *U. maydis* K14 was slightly higher with 0.12 g_ITA_ g_starch_, but 125.6 g l^−1^ of unmetabolized sugar (not accounted in the yield calculation) in form of maltose, maltotriose and maltohexaose remained in the culture supernatant. Hence, further optimization is needed to fully degrade accumulated maltooligomers to glucose, as this appears to be the only sugar efficiently utilized by the strains.

### Analysis of the amylolytic enzyme activity in *Ustilago* species

To optimize the amylolytic activity by metabolic engineering, enzymes present in the secretome needed to be identified and characterized. This is particularly interesting for *U. cynodontis* ITA MAX pH, as this strain is capable of growing on starch. The distribution of sugar mono- and maltooligomers detected during the fermentations showed relatively high levels of glucose, hinting at the exoenzymatic degradation of starch by the secretion of a glucoamylase and/or α-glucosidase. Both enzymes have already been frequently reported for a variety of fungi including for example *A. niger, A. awamori, A. oryzae, Neurospora crassa, Colletotrichum gloeosporioides* (Pandey et al. 2000, Kumar and Satyanarayana 2009). Contrary, endoenzymatic treatment of starch typically results in accumulation of G2 (maltose), G3 (maltotriose), G6 (maltohexaose), and G7 (maltoheptaose) maltooligomers as observed after the α-amylase treatment (Fig. 2D), but was less pronounced during *U. cynodontis* ITA MAX pH fermentation. To test the hypothesis regarding the presence of a glucoamylase and/or α-glucosidase and the absence of an α-amylase in the *U. cynodontis* ITA MAX pH secretome, additional shake flask cultivations were performed on starch, and supernatants were analyzed for their α-amylase activity. Indeed, the culture supernatants did not exhibit any α-amylase activity, confirming the absence or very low activity of this enzyme.

**Figure 3.**
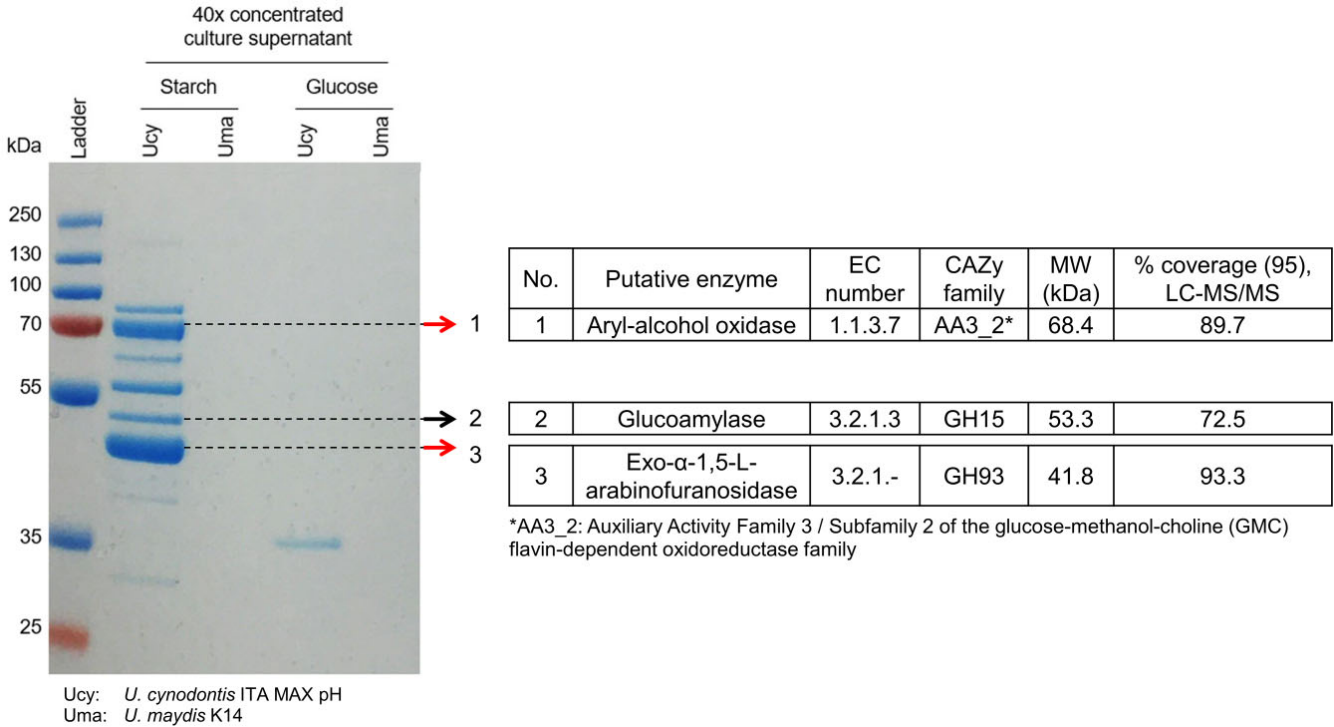
SDS-PAGE and LC-MS/MS analysis of *U. cynodontis* ITA MAX pH and *U. maydis* K14 culture supernatants. Shake flask cultivations were performed in MTM medium containing 15 mM NH_4_Cl, 100 mM MES pH 6.5, and either 10 g l^−1^ gelatinized potato starch or 10 g l^−1^ glucose. Culture supernatants were 40x concentrated using 10 kDa MWCO spin columns and afterwards separated electrophoretically using a 12% polyacrylamide gel. Two bands (indicated by the red arrows) were excised, trypsin-digested, and subjected to LC-MS/MS analysis. The entire supernatant samples were also analyzed via LC-MS/MS. The predicted signal peptide sequences as indicated in Figs S1–S3 are not accounted in the % coverage (95%) values.

Analysis of the secretome via SDS-PAGE and LC-MS/MS exposed a set of extracellular proteins, which are exclusively produced by *U. cynodontis* ITA MAX pH when grown on starch as a sole carbon source (Fig. 3). This confirms the enzymes as starch-induced proteins absent upon cultivation on the conventional feedstock glucose. In contrast to *U. cynodontis* ITA MAX pH, *U. maydis* K14 culture supernatants showed no detectable proteins when incubated with starch, which was in line with the lack of growth on this carbon source (Figs 1B and 3).

Subsequent LC-MS/MS analysis confirmed the presence of a glucoamylase as previously identified by blast analysis (Table 3, Fig. 3, Fig. S1), supporting the initial hypothesis. In contrast, the genome-encoded α-glucosidase (118.0 kDa) was not identified in the secretome via LC-MS/MS. However, a weak protein band could be observed >130 kDa, which might reflect the expected α-glucosidase in its glycosylated form. In addition, a further enzyme belonging to the glycoside hydrolase family 93 (GH93) was detected in high abundance (Fig. 3, Fig. S2). According to the Carbohydrate-Active-enZYmes (CAZy) database, this enzyme shows closest similarity to an exo-α-1,5-l-arabinofuranosidase (Drula et al. 2021). Based on the functional description of a GH93 enzyme in the pythopathogenic fungi *Fusarium graminearum*, this enzyme cleaves l-arabinose side chains from arabinose-substituted oligosaccharides with a strict substrate specificity for linear α-1,5-linked arabinans (Carapito et al. 2009). A similar enzyme activity has been reported for an exo-α-1,5-l-arabinanase (GH93) from both *Chrysosporium lucknowense* C1 (Kühnel et al. 2011) and *Penicillium chrysogenum* 31B (Sakamoto et al. 2004) releasing arabinobiose from the nonreducing end of arabinose oligomers. Since arabinan polymers are an important substitution of hemicellulosic and pectic oligosaccharides in plants (Yeoman et al. 2010), their hydrolysis plays an important role in the complete degradation of the plant cell wall components and is assumed to facilitate enzymatic access of the backbone (Thakur et al. 2019). LC-MS/MS analysis also revealed the presence of an enzyme with 82.3% protein sequence similarity to UMAG_03246 (Fig. 3, Fig. S3). UMAG_03246 was previously detected in *U. maydis* when grown on xylan (Geiser et al. 2013). This enzyme is classified as part of the AA3_2 subfamily of the glucose–methanol–choline flavin-dependent oxidoreductase family. The subfamily also includes UMAG_04044, which is one of the most abundant enzymes in the secretome of *U. maydis* when grown on maize (Couturier et al. 2012). Further characterization identified UMAG_04044 as an aryl-alcohol oxidase (EC 1.1.3.7) with anisyl alcohol, a methoxylated lignin model compound, as the main substrate. This suggests its functional role in lignocellulose deconstruction through lignin degradation, presumably by producing hydrogen peroxide (Hernandez-Ortega et al. 2012, Couturier et al. 2016). Although these latter two secreted enzymes have no clear relation to starch degradation, a concomitant secretion of amylolytic enzymes, the exo-α-1,5-l-arabinofuranosidase as well as the aryl-alcohol oxidase in *U. cynodontis* ITA MAX pH on starch may be associated to its pythopathogenic lifestyle. Lignocellulose-degrading enzymes are supposed to soft or partly degrade the plant cell wall (Doehlemann et al. 2008), which may facilitate access to starch as the primary storage carbohydrate in plants. The potato starch used in this study might retain trace amounts of lignin and heteropolysaccharides from the potato peels, triggering secretion of these putative enzymes (Rodriguez-Martinez et al. 2023). Additional proteins have been detected through SDS-PAGE, but could not be identified due to their low abundance. This may be caused by the minimal expression of plant cell wall-degrading enzymes to prevent triggering an immune response in the plants (Doehlemann et al. 2008). Accordingly, it is reasonable to assume that further cellulases and/or xylanases required for lignocellulose depolymerization are present in the secretome during growth on starch.

Among the identified enzymes—either via LC-MS/MS in the secretome or via tblastn analysis of the *U. cynodontis* genome—the α-amylase, glucoamylase and α-glucosidase are typically related to starch degradation (Vihinen and Mäntsälä 1989). Since no α-amylase activity could be detected, we deleted the glucoamylase and α-glucosidase-encoding genes in the *U. cynodontis* ITA MAX pH to test their involvement in starch hydrolysis.

**Figure 4.**
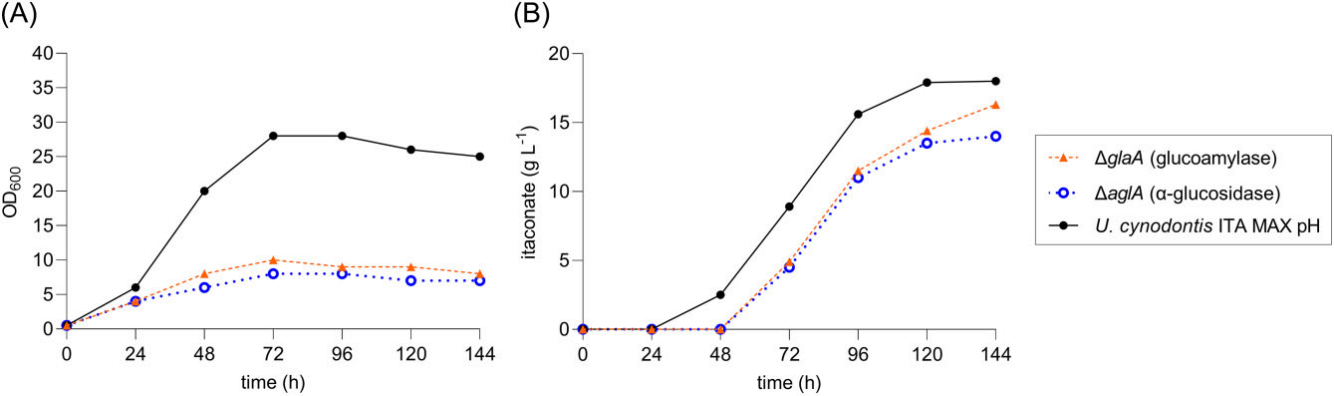
Shake flask cultivations of *U. cynodontis* ITA MAX pH deletion mutants on gelatinized potato starch as a sole carbon source. Shake flask cultivations were performed in MTM medium containing 15 mM NH_4_Cl, 100 mM MES pH 6.5, and 50 g l^−1^ gelatinized potato starch. All modifications were performed in *U. cynodontis* ITA MAX pH. (A) Optical densities and (B) itaconate concentrations throughout the cultivations are shown. The values of a single representative culture are shown.

Remarkably, the knockout of either glucoamylase or α-glucosidase resulted in a reduced, but not abolished growth and itaconate production, thus indicating a redundancy of both enzymes in starch degradation (Fig. 4A and B). It is possible that the accumulation of limit dextrins occurs due to the preferred α-1,4 bond hydrolysis of the α-glucosidase (Lee et al. 2013). Subsequently, glucoamylase may efficiently degrade the remaining α-1,6 linkages. Based on these *in vivo* results and the tblastn analysis (Table 3), the gene with the GenBank accession number CAKMXY010000008 (region: 881 932–885 099) encoding the α-glucosidase is designated as *aglA* and the gene with the GenBank accession number CAKMXY010000014 (region: 83 686–85 185) encoding the glucoamylase is designated as *glaA*. The genes and enzymes are named according to literature convention described in Murphy et al. (2011). Remarkably, the two highly abundant, yet uncharacterized exo-α-1,5-l-arabinofuranosidase (GenBank accession number CAKMXY010000018, region 665 749–665 919, 666 077–666 352, and 666 447–667 148) and aryl-alcohol oxidase (GenBank accession number CAKMXY010000011, region 416 218–416 544 and 416 702–418 270) do not appear to be significantly involved in starch degradation.

**Figure 5.**
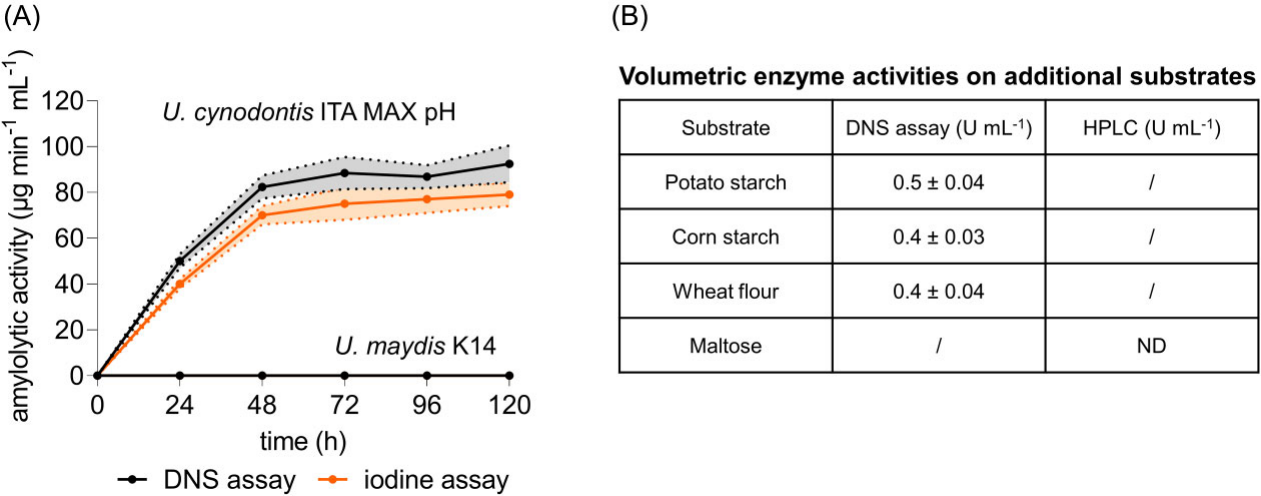
Amylolytic activity in supernatants of *U. cynodontis* ITA MAX pH and *U. maydis* K14 grown on gelatinized potato starch as a sole carbon source. Shake flask cultivations were performed in MTM medium containing 15 mM NH_4_Cl, 100 mM MES pH 6.5, and 10 g l^−1^ gelatinized potato starch. (A) Amylolytic activity in µg min^−1^ ml^−1^ during the cultivation, quantified via DNS (black line) and iodine assay (orange line). (B) Volumetric enzyme activities in U ml^−1^ on potato starch, corn starch, wheat flour, and maltose, calculated based on the molecular weight of glucose. The mean values with standard deviation of three independent biological replicates are shown. ND: not detected;/: not analyzed due to technical limitations like viscosity.

The amylolytic activity of the secretome was determined using gelatinized potato starch as substrate. To this end, starch degradation and reducing sugar accumulation during *U. cynodontis* ITA MAX pH and *U. maydis* K14 cultivation on starch were monitored throughout the entire cultivation via DNS and iodine assays (Fig. 5). These assays enable a differentiation between exoenzymes, such as glucoamylases and α-glucosidases, and endoenzymes, such as α-amylases.

The activity in the *U. cynodontis* ITA MAX pH culture increased almost linearly during the first 48 h, reaching ~82.3 ± 5 µg min^−1^ ml^−1^ as measured by the DNS assay and 70 ± 4 µg min^−1^ ml^−1^ as measured by the iodine assay (Fig. 5A). Nitrogen limitation after 48 h (cf. Fig. 1A) prevented further protein synthesis and led to a stabilization of the amylolytic activity. The activity of the secreted enzymes remained at its maximum level, indicating high stability. Since glucoamylases and α-glucosidases usually lead to a reduction in starch staining capacity along with a significant release of reducing sugars (Glose et al. 1990), the comparable activities measured by both methods serve as a further confirmation for the presence of these two exoenzymes. Contrary, α-amylase activity typically shows up by a rapid decrease in iodine staining capacity with only a small amount of reducing sugars released. Overall, these results clearly reflect the phytopathogenic lifestyle of *Ustilago* species. For bitrophic growth, fungal plant degradation by CAZy needs to be restricted to a minimum level required for penetration (Doehlemann et al. 2008). Higher, unregulated activity would cause severe damage and trigger the plant immune system through sensing of plant cell wall oligomers (Wan et al. 2021), which can be assumed to be also the case for rapid α-amylase-mediated release of starch oligomers. In contrast, exoenzymes like glucoamylases and α-glucosidases were shown to primarily release glucose in a more controlled fashion, thereby probably circumventing strong activation of the immune response.

To allow better comparison with amylolytic activities in other fungi, the volumetric enzyme activities in µmol min^−1^ ml^−1^ (U ml^−1^) were calculated based on the DNS assay using the molecular weight of glucose (Fig. 5B). A maximum enzyme activity of 0.5 ± 0.04 U ml^−1^ was detected at the fifth day after inoculation of *U. cynodontis* ITA MAX pH, which is probably a combination of the activity of both identified exoenzyme. This level of enzymatic activity is consistent with other studies indicating fungal glucoamylase activities between 0.3 U ml^−1^ for culture supernatants (Ogundero and Osunlaja 1986), although much lower than 200 U ml^−1^ for commercially available glucoamylases (Sigma-Aldrich). The activities of α-glucosidases are mostly reported as specific activities in U mg^−1^, which makes the comparison with activities directly measured in culture supernatants without prior enzyme purification difficult.

The hydrolytic activity of the glucoamylase and α-glucosidase was also tested on additional substrates (Fig. 5B). With 0.4 ± 0.03 U ml^−1^ on gelatinized corn starch and 0.4 ± 0.04 U ml^−1^ on gelatinized wheat flower, the activities were comparable to the one observed on potato starch. Interestingly, no activity was determined on maltose, which is one of the preferred substrates of most fungal α-glucosidases (Manjunath et al. 1983, Chiba 1988, 1997). Based on substrate specificity, α-glucosidases can be classified into three main groups. Type-I preferentially degrades heterogeneous linkages (e.g. in sucrose), while types II and III preferentially hydrolyze homogenous linkages (e.g. in maltose, maltooligomers, and starch) with Type-III α-glucosidases being more efficient at degrading polysaccharides such as starch compared to Type-II α-glucosidases (Okuyama et al. 2016). Therefore, it appears that the α-glucosidase present in our culture supernatant is a Type-III α-glucosidase. This is relatively rare among fungal α-glucosidase, as most of them tend to hydrolyze maltose more rapidly than soluble starch (Chiba 1988, Tanaka et al. 2002). To confirm this tendency, degradation of additional maltooligomers with various chain lengths could be tested, which requires prior purification of the glucosidase to prevent interference with the glucoamylase activity. The purified α-glucosidase could also be examined regarding its transglycosylation activity. This activity has been already reported for the α-glucosidase from *Mortierella alliacea*, which could use glycogen and soluble starch to transfer a glycosyl residue to ethanol, thereby producing ethyl α-d-glucopyranoside, a noncariogenic sweetening and flavoring agent (Tanaka et al. 2002).

### Optimization of the amylolytic activity in *U. cynodontis* ITA MAX pH

**Figure 6.**
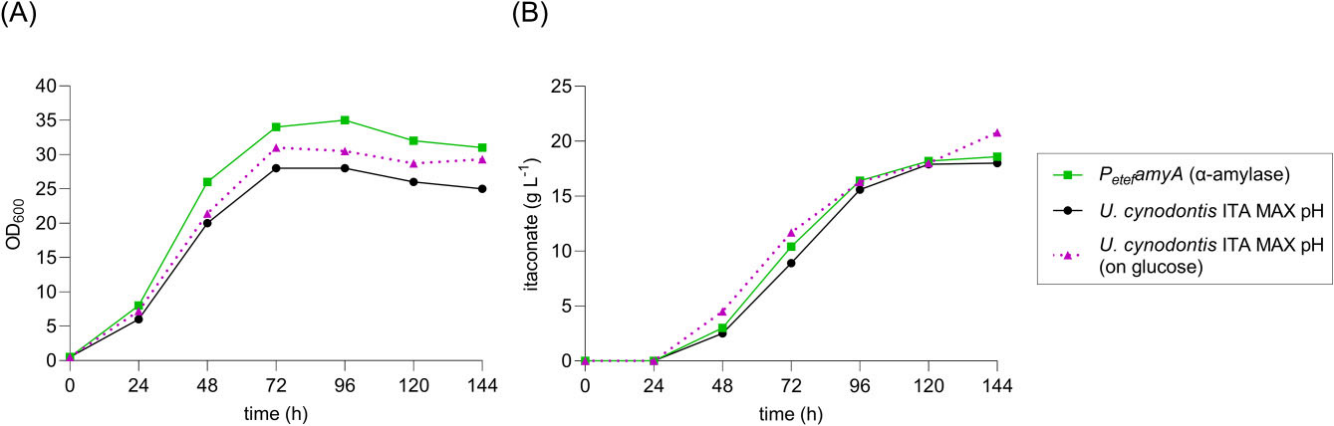
Shake flask cultivations of *U. cynodontis* ITA MAX pH constitutively expressing the native α-amylase gene on gelatinized potato starch as a sole carbon source. Shake flask cultivations were performed in MTM medium containing 15 mM NH_4_Cl, 100 mM MES pH 6.5, and 50 g l^−1^ gelatinized potato starch. (A) Optical densities and (B) itaconate concentrations throughout the cultivations are shown. The values of a single representative culture are shown. The OD_600_ values and itaconate concentrations of *U. cynodontis* ITA MAX pH from Fig. 4 are shown again for comparison. In addition, values form a previous cultivation of *U. cynodontis* ITA MAX pH on 50 g l^−1^ glucose are shown.

The efficient hydrolysis of starch to glucose typically involves the synergetic action of an α-amylase and a glucoamylase. Initially, gelatinized starch is liquefied to maltooligomers, which are then hydrolyzed to glucose by glucoamylases. Since no α-amylase could be detected in *U. cynodontis* ITA MAX pH culture supernatants despite its genomic presence, we constitutively overexpressed the native α-amylase gene with the GenBank accession number CAKMXY010000005 (region: 1 296 343–1 297 953) (Fig. 6).

The constitutive overexpression of the native α-amylase gene in *U. cynodontis* ITA MAX pH significantly improved the growth on starch (Fig. 6A), presumably due to a more efficient starch degradation. This was accompanied by a slightly improved itaconate production compared to the progenitor strain (Fig. 6B). Interestingly, similar KPIs were obtained on glucose and starch during shake flask cultivations, whereas they significantly differed during bioreactor batch fermentations (Fig. 2). This may be due to variations in the C/N ratio, which could be optimized by low cell-density and/or fed-batch fermentations in follow-up studies.

Since native expression of CAZy is expected to be on a low level to minimize plant tissue damage (Doehlemann et al. 2008), conversion of starch to itaconate by *U. cynodontis* ITA MAX pH can likely be further optimized by constitutive overexpression of the native amylolytic genes. This might also enable starch degradation by *U. maydis* K14. In addition, the heterologous overexpression of α-amylases containing a starch-binding domain (Janecek et al. 2003) could be tested for the utilization of raw starch. This would eliminate the energy-intensive initial gelatinization step (Robertson et al. 2006) (cf. Fig. 1), and has already been successfully demonstrated for *A. terreus* (Wong et al. 2007).

## Conclusion

In this work, we investigated the utilization of the low-cost substrate starch by *U. cynodontis* ITA MAX pH and *U. maydis* K14, two *Ustilago* strains that have been previously engineered for efficient itaconate production. *Ustilago cynodontis* ITA MAX pH was able to metabolize gelatinized potato starch, reaching itaconate titers of up 10 g l^−1^ with a yield of ~0.1 g_ITA_ g_starch_^−1^ during respective batch fermentations. This could be traced back to the activity of a glucoamylase and an α-glucosidase in its secretome, which were shown to be synergistically involved in starch degradation. In contrast, *U. maydis* K14 required α-amylase pretreated potato starch hydrolysates for growth and itaconate production. Although the KPIs are yet lower compared to those achieved with glucose in batch fermentations, the utilization of starch has the advantage of causing less osmotic stress. While high concentrations of monomeric glucose at the start of the fermentation typically result in increased osmotic stress of the deeply engineered *Ustilago* strains, continuous enzymatic liberation of single glucose molecules from glucose polymers and their immediate consumption circumvent higher glucose accumulation. However, to exploit the full potential of starch as a substrate for itaconate production, further optimizations such as the constitutive overexpression of the amylolytic genes or the utilization of partly *in situ* hydrolyzed starch are required to achieve higher KPIs. Although further optimization is required, *U. cynodontis* ITA MAX pH has been successfully demonstrated to be a promising host for itaconate production from gelatinized potato starch.

## Supplementary Material

foae023_Supplemental_File

## Data Availability

All data generated or analyzed during this study are included in this published article and its supplementary information files.
