# Supplementary material for: Establishing an itaconic acid production process with Ustilago species on the low-cost substrate starch
Source: FEMS Yeast Res. 2024 Jul 22;24:foae023. doi: 10.1093/femsyr/foae023 (PMC11312366; doi:10.1093/femsyr/foae023)
Supplement: foae023_Supplemental_File [file foae023_supplemental_file.docx]

**Supplementary materials to:**

**Establishing an itaconic acid production process with *Ustilago* species on the low-cost substrate starch**

Philipp Ernst^1^, Astrid Wirtz^1^, Benedikt Wynands^1^, Nick Wierckx^*1^

^1^Institute of Bio- and Geosciences IBG-1: Biotechnology, Forschungszentrum Jülich GmbH, Wilhelm-Johnen-Straße, 52428 Jülich, Germany

*Corresponding author: [n.wierckx@fz-juelich.de](mailto:n.wierckx@fz-juelich.de)

# Supplements


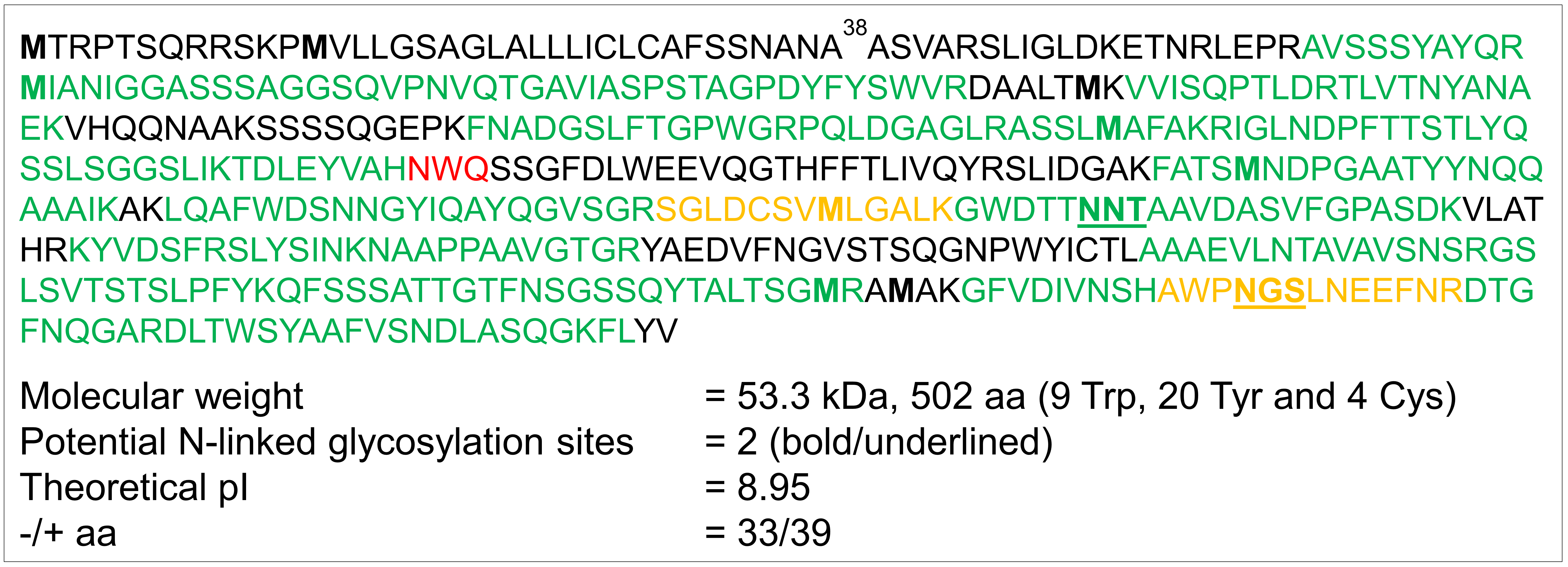


**Figure S1: Amino acid sequence of putative glucoamylase encoded by the gene with the GenBank accession number CAKMXY010000014 (region: 83686 to 85185) in *U. cynodontis* ITA MAX pH identified by LC-MS/MS.** The peptide confidences are ranged by a color code - black: no match; red: 0-50; yellow: 50‑95; green: 95-100. The first 38 aa are predicted as the signal peptide according to Teufel et al. (2022).


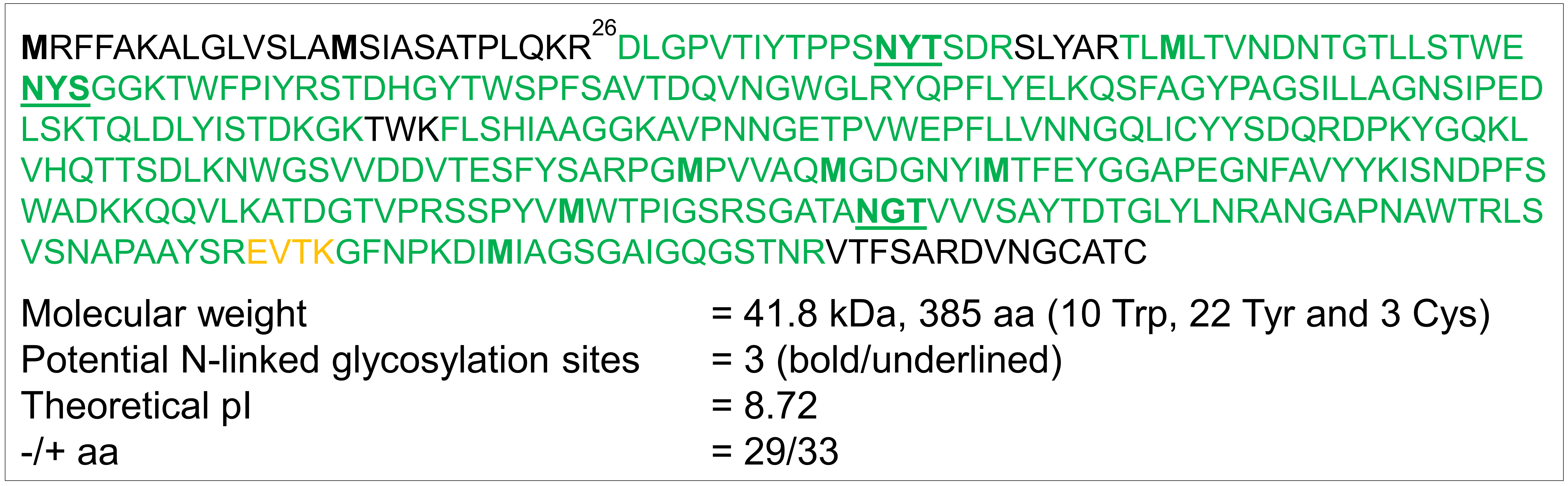


**Figure S2: Amino acid sequence of putative exo-α-1,5-L-arabinofuranosidase encoded by the gene with the GenBank accession number CAKMXY010000018 (region 665749 to 665919, 666077 to 666352, and 666447 to 667148) in *U. cynodontis* ITA MAX pH identified by LC-MS/MS.** The peptide confidences are ranged by a color code - black: no match; red: 0-50; yellow: 50‑95; green: 95-100. The first 26 aa are predicted as the signal peptide according to Teufel et al. (2022).


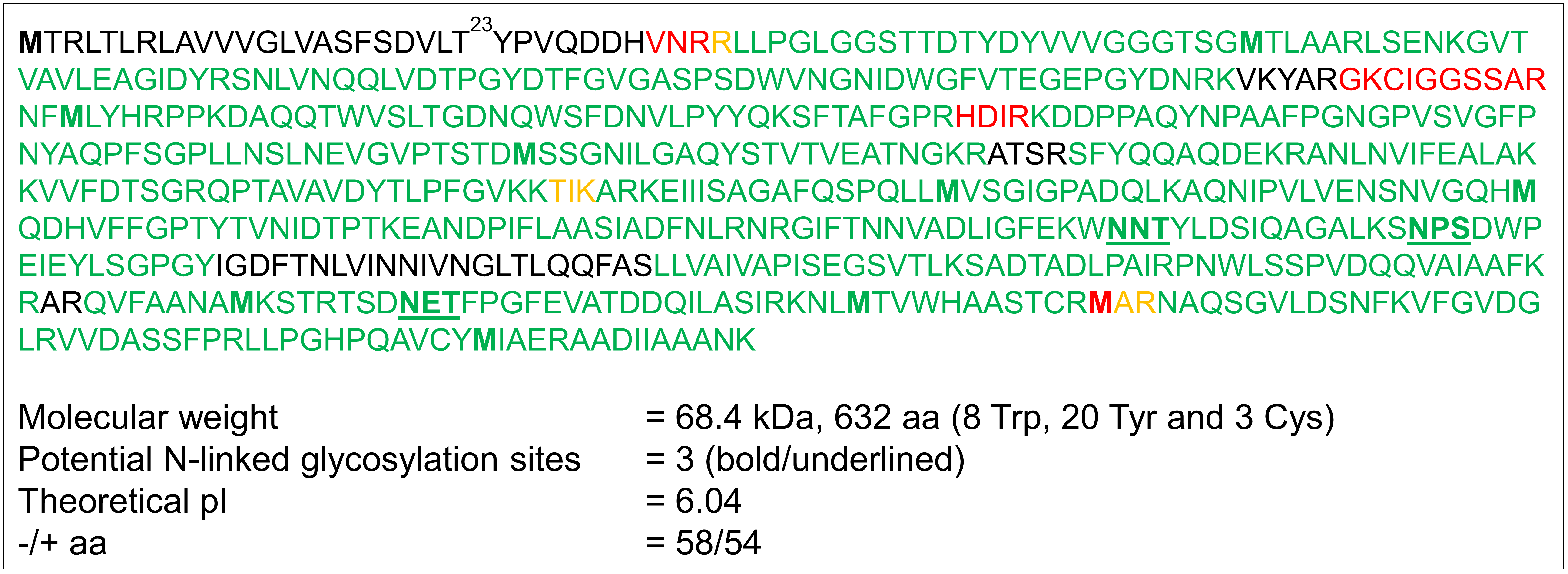


**Figure S3: Amino acid sequence of putative aryl-alcohol oxidase encoded by the gene with GenBank accession number CAKMXY010000011 (region 416218 to 416544 and 416702 to 418270) in U. cynodontis ITA MAX pH identified by LC‑MS/MS.** The peptide confidences are ranged by a color code - black: no match; red: 0-50; yellow: 50‑95; green: 95-100. The first 23 aa are predicted as the signal peptide according to Teufel et al. (2022).

**Table S1: Oligonucleotides used for the generation of deletion and overexpression constructs.**

| **Primer name** | **Sequence (5’-3’) and description** |
| --- | --- |
| PE1_fwd | gttcttctaggatcactctcggcatggac  Amplification of pJET1.2 backbone for the generation of *U. cynodontis* α-amylase overexpression construct |
| PE2_rev | gcgagacgaagataataatggtttcttagacgtcag  Amplification of pJET1.2 backbone for the generation of *U. cynodontis* α-amylase overexpression construct |
| PE3_fwd | cattattatcttcgtctcgcgcgtttcg  Amplification of the *P_etef_* promotor for the generation of *U. cynodontis* α-amylase overexpression construct |
| PE4_rev | aggaagccatgatcccgtggatgatgttgtc  Amplification of the *P_etef_* promotor for the generation of *U. cynodontis* α-amylase overexpression construct |
| PE5_fwd | ccacgggatcatggcttcctctgccaac  Amplification of the α-amylase gene for the generation of *U. cynodontis* α-amylase overexpression construct |
| PE6_rev | gagagtgatcctagaagaactgacggtgc  Amplification of the α-amylase gene for the generation of *U. cynodontis* α-amylase overexpression construct |
| PE7_fwd | cagactgactggatctttctagaagatctcctac  Amplification of pJET1.2 backbone for the generation of UMAG_04064 deletion construct |
| PE8_rev | tgaattgattctatcttgctgaaaaactcg  Amplification of pJET1.2 backbone for the generation of UMAG_04064 deletion construct |
| PE9_fwd | ttttcagcaagatagaatcaattcaagcggtg  Amplification of 5’-UTR flank for the generation of UMAG_04064 deletion construct |
| PE10_rev | ggcaagcttcgcgggcttcaagatcgtggttaac  Amplification of 5’-UTR flank for the generation of UMAG_04064 deletion construct |
| PE11_fwd | gatcttgaagcccgcgaagcttgccggcag  Amplification of G418R cassette for the generation of UMAG_04064 deletion construct |
| PE12_rev | aagtggagatatcgccgcactcctacagcttg  Amplification of G418R cassette for the generation of UMAG_04064 deletion construct |
| PE13_fwd | taggagtgcggcgatatctccacttgccgg  Amplification of 3’-UTR flank for the generation of UMAG_04064 deletion construct |
| PE14_rev | cttctagaaagatccagtcagtctgtcagtc  Amplification of 3’-UTR flank for the generation of UMAG_04064 deletion construct |
| PE15_fwd | tggttttgcctaatctttctagaagatctcctac  Amplification of pJET1.2 backbone for the generation of UMAG_02740 deletion construct |
| PE16_rev | ttgcgatgcttctatcttgctgaaaaactcg  Amplification of pJET1.2 backbone for the generation of UMAG_02740 deletion construct |
| PE17_fwd | tttcagcaagatagaagcatcgcaacgcaag  Amplification of 5’-UTR flank for the generation of UMAG_02740 deletion construct |
| PE18_rev | gcaagcttcgcgaagattcccacgatgctataac  Amplification of 5’-UTR flank for the generation of UMAG_02740 deletion construct |
| PE19_fwd | tcgtgggaatcttcgcgaagcttgccggcag  Amplification of G418R cassette for the generation of UMAG_02740 deletion construct |
| PE20_rev | ggcctcctccaaggccgcactcctacagcttg  Amplification of G418R cassette for the generation of UMAG_02740 deletion construct |
| PE21_fwd | taggagtgcggccttggaggaggcccaatc  Amplification of 3’-UTR flank for the generation of UMAG_02740 deletion construct |
| PE22_rev | cttctagaaagattaggcaaaaccaagaacatgc  Amplification of 3’-UTR flank for the generation of UMAG_02740 deletion construct |
